# Intensified seed spices-based cropping systems for higher productivity, resource-use efficiency, soil fertility and profitability in arid and semi-arid regions of India

**DOI:** 10.1371/journal.pone.0292955

**Published:** 2023-10-18

**Authors:** Narendra Chaudhary, Shiv Lal, Ravindra Singh, M. D. Meena, S. S. Meena, R. D. Meena, C. K. Jangir, V. Bhardwaj, Asheesh Sharma

**Affiliations:** 1 Indian Council of Agricultural Research-National Research Centre on Seed Spices, Ajmer, Rajasthan, India; 2 College of Agriculture, Jawaharlal Nehru Krishi Vishwa Vidyalaya, Narmadapuram, Madhya Pradesh, India; Central Arid Zone Research Institute, INDIA

## Abstract

Coriander, fenugreek, nigella etc. are collectively known as seed spices. They are “*High value and low volume crops*” and considered cash crops for the growers of arid and semi-arid regions of India. Coriander, fenugreek and nigella are grown during the *rabi* season and take hardly 130–140 days to attain full maturity. In this context, farmers are not able to develop income from available arable land round the year, even though they have sufficient resources as well as manpower. Therefore, coriander, fenugreek and nigella-based cropping systems, four of each (total 12) were evaluated during 3 consecutive years (2019–20 to 2021–22) for their productivity, resource-use efficiency, economics and soil fertility. The results showed that among the seed spices-based cropping systems, maximum system productivity (5193 kg ha^-1^), production efficiency (18.81 kg ha^-1^ day^-1^), water-use efficiency (2.31 kg ha^-1^ mm^-1^), economic efficiency (11.85 US $ ha^-1^ day^-1^), net return (3270 US $ ha^-1^), benefit:cost ratio (3.27) and available N (165.6 kg ha^-1^) were observed under nigella-green coriander-mungbean cropping system. Hence, seed spices growers are recommended to adopt nigella-green coriander-mungbean cropping system in order to realize better productivity, resource-use efficiency, soil fertility and profitability.

## Introduction

The seed spices constitute an important group of agricultural commodities and play a significant role in the national economy. Coriander, cumin, fennel, fenugreek, ajwain, nigella, dill, celery, anise, caraway are known as seed spices. They are grown in an area of 1.92 million hectares with annual production of 1.91 million tonnes. Despite the continuance of COVID-19 pandemic during 2021–22, spices export from India continued its upward trend [1]. India has always been recognized as a land of spices [2]. Globally, India is the largest producer, consumer and exporter of seed spices. They are considered “*High value and low volume crops*” with lifeline for the growers of arid and semi-arid regions of India as these crops are considered cash crop [3]. Seed spices are low-input loving and short-duration crops. Coriander, fenugreek and nigella are mostly cultivated in arid and semi arid regions of India during the *rabi* season and take hardly 130–140 days to attain the full maturity. In this context, farmers are not able to develop income/output from available arable land round the year, even though they have enough resources as well manpower. They do not have alternate option for a choice of crops to grow during summer and *kharif* season as these regions are prone to extreme hot during summer and low rainfall (average~500 mm) during *kharif*. So, the survival of crops itself is a big challenge. Under such circumstances, rural youth keep on migrating towards the urban area in search of employment which eventually results in poor agricultural productivity owing to a lack of manpower.

In India, agriculture and allied sectors share 21.1% of total gross value added (GVA) during 2022–23 and employing 54.6% of the population in 2011. During 2019–20 out of 328 only 140 million hectares is cultivated in the country, resulting lower cropping intensity of 151% The increasing population and decreasing average farm size to 1.17 ha in India, resulting in 86% of the total 119 million farmers becoming marginal (upto 1ha) and small (1–2 ha) [4]. The viability of the marginal and small farmers is a major challenge for Indian agriculture, calling for a sustainable cropping system to earn economically viable livelihoods [5]. In the era of dwindling land, water and energy-use efficiency are important for the suitability of cropping system [6]. Hence, the selection of crops needs to be planned to utilize synergism among crops towards the efficient utilization of resources and to increase overall productivity [7]. Seed spices being cash crop cannot be replaced by other crops during *rabi* season and can be grown with limited resources to realize maximum returns. Growing crops such as fruits, vegetables [8], pulses and oilseeds [9] is an alternative approach for realizing overall higher productivity and profitability. The inclusion of such crops along with fodder will help to improve the economic situation of small and marginal farmers [10] and fulfil the demand for vegetables, fruits, pulses and fodder. High yielding, short-duration and biotic & abiotic resistant legumes and vegetable crops have the best chance of acceptance by the farmers for their ability to fit into the existing seed spices based system and to maintain or improve the short and long term productivity and economic viability of the system. Legume crops fix atmospheric N, enrich soil fertility, and could help to sustain the long-term productivity of seed spices-based cropping systems. The legumes in rotation are not only responsible for biological nitrogen fixation, but they also improve nutrient availability, soil structure, reduce disease incidence and promote mycorrhizal colonization [11]. It has also been observed that the inclusion of a third crop of legume during the summer season proved better in enhancing the productivity of the sequence [12]. Legumes play an important role in this region in the view of low soil fertility, as atmospheric N fix by these crops may fulfil the nutrition requirement of succeeding crop in cropping system. Mungbean, clusterbean and fenugreek are the potential legumes for this region. Other than seed, clusterbean fresh pods and fenugreek leaves may also be used as vegetables. Clusterbean stalks and leaves may also fulfil fodder requirement for livestock after harvesting fresh pods. Hence, efforts are being made to promote the cultivation of seed spices for livelihood security during *rabi* with other suitable crops during summer and *kharif* seasons in sequence in order to sustain productivity and resource-use efficiency. Therefore, this study was carried out to determine the most productive, resource-use efficient, remunerative and profitable cropping system among the seed spices-based cropping systems in arid and semi-arid regions of India.

## Materials and methods

### Experimental details

A field experiment on intensification of seed spices-based cropping systems was conducted for three consecutive years (2019–20 to 2021–22) at the Research Farm of Indian Council of Agricultural Research-National Research Centre on Seed Spices, Ajmer, India. The Centre lies on 74° 35’ 39’’ E to 74° 36’ 01’’ E longitude and 26° 22’ 12’’ N to 26° 22’ 31’’ N latitude at an altitude of 460.17 m above mean sea level. The rainfall in the area is highly erratic and more than 90% of the rain is received from July to September with several intermittent long dry spells. The average rainfall is ~500 mm. The temperature ranges from 2–5°C during January and 42–45°C during May. The meteorological data showed a marked variation in weather conditions during the three years of experimentation (S1 Table). The minimum temperature ranged from 6.13 to 25.92°C with the mean of around 18°C during three years. The maximum temperature varied from 21.93 to 38.37°C with of around 32°C during three years. Mean evaporation during three years was 7.38 mm/day. The pattern of rainfall varied significantly during the three years. In 2019–20, 2020–21 and 2021–22 rainfall received were around 262, 850 and 926 mm respectively. Maximum rainfall received during south-west monsoon period i.e. June to September. However during 2020 maximum rainfall (160 mm) received in October month which coincided with the sowing time of *rabi* season crops Total rainfall received was around 2038 mm during three years.

The treatments consisting of 12 cropping system were laid out in randomized block design (RBD) with three replications of plot size of 3 x 3 m. The cropping systems (CS) were:

CS_1_–Coriander (*Coriandrum sativum* L.)-clusterbean (*Cyamopsis tetragonoloba* L.)-mungbean
CS_2_–Coriander-clusterbean-clusterbean
CS_3_–Coriander-green coriander (*Coriandrum sativum* L.)-clusterbean
CS_4_–Coriander-green coriander-mungbean (*Vigna radiata*)
CS_5_–Fenugreek (*Trigonella foenum-graecum* L.)-clusterbean-mungbean
CS_6_–Fenugreek-clusterbean-clusterbean
CS_7_–Fenugreek-green coriander-clusterbean
CS_8_–Fenugreek-green coriander-mungbean
CS_9_–Nigella (*Nigella sativa* L.)-clusterbean-mungbean
CS_10_–Nigella-clusterbean-clusterbean
CS_11_–Nigella-green coriander-clusterbean
CS_12_–Nigella-green coriander-mungbean

The details of the varieties and agronomic practices followed during field experimentation are given in S2 Table. During *rabi* season coriander, fenugreek and nigella were grown for seed purposes. During summer season, green coriander was grown under 50% green shade net to protect the crop from extreme heat and used for leaf purposes. Summer season clusterbean was used for seed purpose. *Kharif* season mungbean and clusterbean were also used for seed purpose. Economic yields of the component crops were converted to coriander equivalent yield (CEY) on price basis in order to compare the performance of cropping systems by taking into account the prevailing market price of coriander (Rs. 66 kg^-1^), fenugreek (Rs. 50 kg^-1^), nigella (Rs. 200 kg^-1^), green coriander (Rs. 200 kg^-1^), clusterbean (Rs. 60 kg^-1^) and mungbean (Rs. 80 kg^-1^). The total field duration of a cropping system expressed in a percentage of 365 days was taken as the land-use efficiency of the system [13]. Production efficiency was expressed as the ratio of system productivity (kg CEY ha^-1^) to the total duration of the system in days [14]. Water-use efficiency was computed by dividing system productivity by total amount of water applied in the system and expressed as kg CEY ha^-1^ mm^-1^. (cropping system; no. of irrigations; amount of water applied in mm), (CS_1_; 14; 1746), (CS_2_; 16; 1676), (CS_3_; 19; 2176), (CS_4_; 19; 2246), (CS_5_; 12; 1673), (CS_6_; 12; 1603), (CS_7_; 17; 2103), (CS_8_; 17; 2173), (CS_9_; 14; 1746), (CS_10_; 14; 2009), (CS_11_; 19; 2176), (CS_12_; 19; 2246). Economic efficiency is calculated by dividing net returns by the total duration of the system.

### System productivity

The productivity of different crop sequences was compared by calculating their economic coriander equivalent yield (CEY) by using the formula given by Ahlawat and Sharma [15].


$$\text{CEY}\;\left({\text{kg}\;\text{ha}}^{-1}\right)=\frac{\text{Yield}\;\text{of}\;\text{each}\;\text{crop}\;\left({\text{kg}\;\text{ha}}^{-1}\right)\times \text{Economic}\;\text{value}\;\text{of}\;\text{respective}\;\text{crop}\;\left({\text{Rs}\;\text{kg}}^{-1}\right)}{\text{Price}\;\text{of}\;\text{coriander}\;\text{seed}\;\left({\text{Rs}\;\text{kg}}^{-1}\right)}$$ (1)

System productivity of each system was calculated by summation of the CEY of each crop grown in *rabi*, summer and *kharif* season.

### Soil analysis

To study the changes in soil fertility, initial soil samples were collected with an auger from 0–15 cm soil depth at 10 locations in the experimental area as per standard procedure. The samples were thoroughly mixed, dried and passed through a 100 mesh sieve and kept in poly bags for analysis of organic carbon, available N (Alkaline permanganate method), available P (0.5 N NaHCO_3_ extractable) and available K (Flame photometric method). After completion of three crop cycles, soil samples were again collected from 3 locations of each cropping system for analysis of above said parameters.

### Economic analysis

The average price of individual input and output during the experimental period were taken into the account. The cost of cultivation, calculated on existing input cost and economic value of different crop produce, based on market price (Indian rupees, 1 Rs = 0.012 US $). Net return or profit was calculated by subtracting the cost of cultivation from the gross return of the produce. The benefit:cost ratio was worked out by dividing gross return by the cost of cultivation for various cropping systems.

### Statistical analysis

The data were subjected to analysis of variance (ANOVA) by R software, version 4.3.0. The least significant difference (LSD) test at a significance level of *p* <0.05 was performed to compare the means, if the f-value was significant.

## Results

### System productivity

The sum of three seasons of CEY is represented as system productivity (Table 1). System productivity differed significantly among the seed spices-based cropping systems. The maximum system productivity recorded in nigella-green coriander-mungbean (5193 kg ha^-1^) followed by nigella-green coriander-clusterbeanbean (4783 kg ha^-1^). Fenugreek-green coriander-clusterbean produced third highest system productivity (4572 kg ha^-1^). The other cropping systems were followed the order (kg ha^-1^): nigella-clusterbean-clusterbean (3834) > fenugreek- clusterbean-clusterbean & coriander-green coriander-clusterbean (3350) > nigella-clusterbean-mungbean (3105) > coriander-green coriander-mungbean (2873) > coriander-clusterbean-clusterbean (2596) > fenugreek-clusterbean-mungbean (2595) > fenugreek-green coriander-mungbean (2525) > coriander-clusterbean-mungbean (2279).

**Table 1:** Economic yields of component crops, season productivity and system productivity of various seed spices-based cropping systems (Pooled over data of three years).

Cropping system	Economic yield (kg ha^-1^)			Season productivity (CEY kg ha^-1^)			System productivity (CEY kg ha^-1^)
	*Rabi*	Summer	*Kharif*	*Rabi*	Summer	*Kharif*	
Coriander-clusterbean-mungbean	586.8	1534	245.6	586.8	1394	297.6	2279^e^
Coriander-clusterbean-clusterbean	611.6	1317	865.8	611.6	1197	787.0	2596^de^
Coriander-green coriander-clusterbean	812.7	602.3	783.3	812.7	1825	712.1	3350^cde^
Coriander-green coriander-mungbean	737.1	622.5	206.4	737.1	1886	250.2	2873^de^
Fenugreek-clusterbean-mungbean	1276	1490	226.2	967.3	1354	274.1	2595^de^
Fenugreek-clusterbean-clusterbean	1387	1611	917.5	1051	1465	834.1	3350^cde^
Fenugreek-green coriander-clusterbean	1620	872.6	770.9	1227	2644	700.7	4572^abc^
Fenugreek-green coriander-mungbean	1514	373.7	202.7	1147	1132	245.7	2525^e^
Nigella-clusterbean-mungbean	462.2	1634	180.0	1400	1486	218.1	3105^de^
Nigella-clusterbean-clusterbean	601.7	1372	840.6	1823	1247	764.1	3834^bcd^
Nigella-green coriander-clusterbean	542.3	802.0	781.2	1643	2430	710.2	4783^ab^
Nigella-green coriander-mungbean	628.7	992.3	231.9	1905	3007	281.0	5193^a^
LSD (*p* < 0.05)							1294
Level of significance							***

Means with different letters are significantly different within columns (LSD test, *p* < 0.05). Values are means of three years of study.

***, **, * and NS indicate *p* < 0.001, *p* < 0.01, *p* < 0.05 and *p* > 0.05, respectively.

CEY, coriander equivalent yield

### Resource-use efficiency

The highest LUE registered in coriander-clusterbean-clusterbean and nigella-clusterbean-clusterbean (85.47%, Table 2) rotations that utilized the land more efficiently than other cropping systems. The maximum production efficiency (18.81 kg ha^-1^ day^-1^), water-use efficiency (2.31 kg ha^-1^ mm^-1^) and economic efficiency (11.85 US $ ha^-1^ day^-1^) were recorded in the nigella-geen coriander-mungbean sequence which proved significantly superior over the rest of the sequences. Nigella-green coriander-clusterbean ranked second in terms of production efficiency (15.73 kg ha^-1^ day^-1^), water-use efficiency (2.19 kg ha^-1^ mm^-1^) and economic efficiency (9.84 US $ ha^-1^ day^-1^), whereas third highest production (15.50 kg ha^-1^ day^-1^), water-use (2.17 kg ha^-1^ mm^-1^) and economic (9.51 US $ ha^-1^ day^-1^) efficiency observed under fenugreek-green coriander-clusterbean cropping system. Notwithstanding, the coriander-clusterbean-mungbean gave significantly lowest production and economic efficiency. Lowest water-use efficiency recorded with fenugreek-green coriander-mungbean.

**Table 2:** Resource-use efficiency of various seed spices-based cropping systems (Pooled over data of three years).

Cropping system	Land-use efficiency (%)	Production efficiency (kg CEY ha^-1^ day^-1^)	Water-use efficiency (kg CEY ha^-1^ mm^-1^)	Economic efficiency (US $ ha^-1^ day^-1^)
Coriander-clusterbean-mungbean	80.00^d^	8.02^d^	1.30^cd^	2.43^c^
Coriander-clusterbean-clusterbean	85.47^a^	8.32^d^	1.54^bcd^	2.80^c^
Coriander-green coriander-clusterbean	81.09^c^	11.02^cd^	1.53^bcd^	6.13^bc^
Coriander-green coriander-mungbean	75.61^g^	10.41^d^	1.27^cd^	5.49^c^
Fenugreek-clusterbean-mungbean	77.53^f^	9.43^d^	1.55^bcd^	3.02^c^
Fenugreek-clusterbean-clusterbean	83.01^b^	11.05^cd^	2.09^ab^	4.66^c^
Fenugreek-green coriander-clusterbean	78.63^e^	15.50^abc^	2.17^a^	9.51^ab^
Fenugreek-green coriander-mungbean	73.15^f^	9.45^d^	1.16^d^	3.47^c^
Nigella-clusterbean-mungbean	80.00^d^	10.93^d^	1.77^abc^	4.16^c^
Nigella-clusterbean-clusterbean	85.47^a^	12.29^bcd^	1.90^ab^	5.50^c^
Nigella-green coriander-clusterbean	81.09^c^	15.73^ab^	2.19^a^	9.84^ab^
Nigella-green coriander-mungbean	75.61^g^	18.81^a^	2.31^a^	11.85^a^
LSD (*p* < 0.05)	0.83	4.54	0.60	3.97
Level of significance	***	***	***	**

Means with different letters are significantly different within columns (LSD test, *p* < 0.05).

***, **, * and NS indicate *p* < 0.001, *p* < 0.01, *p* < 0.05 and *p* > 0.05, respectively.

Values are means of three years of study. CEY, coriander equivalent yield

### Economics

The net return and benefit:cost ratio of seed spices-based cropping systems differed significantly among themselves (Table 3). In spite of highest cost of cultivation, nigella-green coriander-mungbean realized the highest net return (3270 US $ ha^-1^) followed by nigella-green coriander-clusterbean (2913 US $ ha^-1^). The higher market price of nigella and green coriander (2.42 US $ kg^-1^ of each) along with greater productivity reinforced these systems to gain more profit in comparison to other cropping systems. The fenugreek-green coriander-clusterbean sequence (2730 US $ ha^-1^) proved third most remunerative cropping system. Lowest net return (711 US $ ha^-1^) was recorded with coriander-clusterbean-mungben sequence. With respect to benefit:cost ratio, six most profitable cropping systems followed the order: nigella-green coriander-mungbean (3.27) > nigella-green coriander-clusterbean (3.14) > fenugreek-green coriander-clusterbean (2.92) > coriander-green coriander-clusterbean (2.53) > coriander-green coriander-mungbean (2.36) > nigella-clusterbean-clusterbean (2.29). Lowest benefit:cost ratio was found under coriander-clusterbean-mungbean (1.65). The lower market price of coriander, cluster bean and mungbean accompanied with low system productivity marked the system economically unviable.

**Table 3:** Economic analysis of various seed spices-based cropping systems (Pooled over data of three years).

Cropping system	Gross return (US $ ha^-1^)	Cost of cultivation (US $ ha^-1^)	Net return (US $ ha^-1^)	Benefit:cost ratio
Coriander-clusterbean-mungbean	1795	1084	711^d^	1.65^e^
Coriander-clusterbean-clusterbean	2046	1172	874^d^	1.74^de^
Coriander-green coriander-clusterbean	2992	1175	1817^bcd^	2.53^abc^
Coriander-green coriander-mungbean	2627	1111	1516^d^	2.36^bcd^
Fenugreek-clusterbean-mungbean	2056	1201	855^d^	1.71^de^
Fenugreek-clusterbean-clusterbean	2654	1239	1415^d^	2.14^bcde^
Fenugreek-green coriander-clusterbean	4145	1415	2730^abc^	2.92^ab^
Fenugreek-green coriander-mungbean	2225	1297	928^d^	1.71^de^
Nigella-clusterbean-mungbean	2459	1242	1217^d^	1.98^cde^
Nigella-clusterbean-clusterbean	3037	1321	1716^cd^	2.29^bcd^
Nigella-green coriander-clusterbean	4270	1357	2913^ab^	3.14^a^
Nigella-green coriander-mungbean	4708	1438	3270^a^	3.27^a^
LSD (*p* < 0.05)			1129	0.66
Level of significance			***	***

Means with different letters are significantly different within columns (LSD test, *p* < 0.05).

***, **, * and NS indicate *p* < 0.001, *p* < 0.01, *p* < 0.05 and *p* > 0.05, respectively.

Values are means of three years of study.

### Soil fertility status

A perusal of data (Table 4) showed that cropping systems differed significantly concerning soil fertility. A significant increase in organic carbon was recorded in all the seed spices-based cropping systems except nigella-clusterbean-mungbean over the initial soil-test value. The maximum increase in organic carbon was observed in fenugreek-green coriander-clusterbean (0.62%), which was at par with fenugreek-clusterbean-clusterbean in comparison to initial organic carbon (0.23%). Nonetheless, the most productive sequences *viz*. nigella-green coriander-mungbean and nigella-green coriander-clusterbean could register 0.57 and 0.55% organic carbon respectively. The available N content improved significantly in all the cropping systems over the initial available N content. The highest increase was observed in nigella-green coriander-mungbean and fenugreek-green coriander-clusterbean (165.6 kg ha^-1^), followed by nigella-green coriander-clusterbean (164.3 kg ha^-1^) over initial value (110 kg ha^-1^). Available P content decreased significantly from their initial value. Among the cropping system significantly higher available P was recorded under the coriander-green coriander-clusterbean (9.01 kg ha^-1^) followed by fenugreek-green coriander-mungbean (8.74 kg ha^-1^). Available K content was also depleted in all the cropping systems over the initial value. However, significantly higher available K content was registered under coriander-green coriander-clusterbean (222.8 kg ha^-1^) followed by coriander-green coriander-mungbean (219.1 kg ha^-1^).

**Table 4:** Chemical properties of soil after completion of three years of experimentation.

Cropping system	Available nutrients (kg ha^-1^)			
	Organic carbon (%)	N	P	K
Coriander-clusterbean-mungbean	0.50^bc^	138.4^de^	8.67^c^	209.4^de^
Coriander-clusterbean-clusterbean	0.43^cd^	153.5^bc^	8.50^c^	215.4^cd^
Coriander-green coriander-clusterbean	0.54^ab^	142.6^cde^	9.01^b^	222.8^b^
Coriander-green coriander-mungbean	0.51^b^	141.7^de^	8.61^c^	219.1^bc^
Fenugreek-clusterbean-mungbean	0.43^cd^	132.1^e^	8.44^cd^	206.4^e^
Fenugreek-clusterbean-clusterbean	0.62^a^	137.9^de^	7.88^e^	210.6^de^
Fenugreek-green coriander-clusterbean	0.62^a^	165.6^a^	8.53^c^	211.0^de^
Fenugreek-green coriander-mungbean	0.37^d^	148.9^cd^	8.74^bc^	214.4^cd^
Nigella-clusterbean-mungbean	0.24^e^	138.8^de^	7.46^f^	210.5^de^
Nigella-clusterbean-clusterbean	0.35^d^	153.9^bc^	7.95^e^	198.0^f^
Nigella-green coriander-clusterbean	0.55^ab^	164.3^ab^	8.14^de^	210.5^de^
Nigella-green coriander-mungbean	0.57^ab^	165.6^a^	7.92^e^	211.0^de^
Initial soil-test values	0.23^e^	110.0^f^	11.5^a^	251.0^a^
LSD (*p* < 0.05)	0.079	10.74	0.31	6.61
Level of significance	***	***	***	***

Means with different letters are significantly different within columns (LSD test, *p* < 0.05).

***, **, * and NS indicate *p* < 0.001, *p* < 0.01, *p* < 0.05 and *p* > 0.05, respectively.

## Discussion

Among the seed spices-based cropping systems, sequences involving nigella, green coriander and legume produced significantly higher system productivity in comparison to those involving either two or three legumes in sequences or followed monoculture. The higher system productivity of nigella-green coriander-mungbean and nigella-green coriander-clusterbean cropping systems reflected the residual advantage of a legume crop on the succeeding nigella crop besides contribution in total system productivity. Likewise, Singh *et al*. [16] reported higher REY (rice-equivalent yield) when legumes were included in the sequence. Further, higher yield level of green coriander [17] along with the higher market price of nigella and green coriander also proved these systems superior in terms of productivity over the other systems. During off-season (summer) green coriander for leaf purpose fetched a higher market price, due to non-availability in the market as coriander is mainly grown in *rabi* season. Green coriander in off-season was economically remunerative. The mungbean and clusterbean in nigella-green coriander-mungbean and nigella-green coriander-clusterbean respectively markedly contributed to the system besides enhancing the productivity of succeeding nigella crop and consequently resulted in higher system productivity. The introduction of a legume crop in the cropping system may have advantages of nitrogen addition through biological nitrogen fixation [18] including nutrient recycling from deeper soil layers, minimising soil compaction, organic matter inputs to soil, breaking the weed and pest cycles and minimize the possible harmful allelopathic effects [19]. These results support earlier studies [20, 21], which advocated that the addition of a third crop as a legume in the sequence resulted in higher yield and profitability [22, 23]. Legumes are known to offer a special advantage regarding the stability of the system because of their effect on soil nitrogen balance and wider adaptability to diverse conditions [24]. When used in cropping systems, it is often supposed that legumes will satisfy a large part of their own nitrogen requirement through biological N fixation, spare soil N shared with non-legume crops, this benefits the subsequent crops [25]. In the present study, the system with continuous two legumes *viz*. coriander-clusterbean-mungbean; coriander-clusterbean-clusterbean; fenugreek-green coriander-mungbean; nigella-clusterbean-mungbean; nigella-clusterbean-clusterbean (except fenugreek-green coriander-clusterbean) or three legumes *viz*. fenugreek-clusterbean-mungbean; fenugreek-clusterbean-clusterbean in sequence exhibited lower system productivity. This might be attributed to the biological N fixed by the preceding legume could not be utilized by the succeeding legume. The cropping systems *viz*. coriander-green coriander-clusterbean and coriander-green coriander-mungbean were not performed upto the mark eventhough green coriander was there in the sequences. This might be attributed to monoculture (coriander-green coriander) reflected as low yield of succeeding green coriander crop in comparison to other sequence having green coriander as summer crop. Monoculture may reduce nutrient-use efficiency and soil functional micro-organisms [26] resulting in lower productivity. Intensification of crop sequences with legumes has been well known to increase yield [27–29]. The variation in the economic yield of crops might be due to variations in weather parameters (rainfall and its distribution and temperature) and improvement in soil available N due to the inclusion of legume crops in sequences.

Land-use efficiency was higher with the systems included clusterbean followed by clusterbean among all the cropping systems. This might be due to more field occupancy of clusterbean over green coriander and mungbean in the summer and *kharif* season respectively [30]. Production efficiency, water-use efficiency and economic efficiency followed the same trend as of system productivity and recorded highest in nigella-green coriander-mungbean closely followed by nigella-green coriander-clusterbean cropping system.

Net returns are directly linked to the price that the grower receives for the produce and inversely related to the cultivation cost. Among seed spices-based cropping systems, nigella-green coriander-mungbean was found the most profitable cropping system followed by nigella-green coriander-clusterbean. Both net return and benefit:cost ratio were recorded highest under nigella-green coriander-mungbean followed by nigella-green coriander-clusterbean cropping system. This could be attributed to higher market price of both nigella and green coriander together with higher production level of green coriander. Biswas *et al*. [31] reported that higher production potential of potato resulted in greater benefit:cost ratio. Reduced supply of market and greater demand of consumers for green coriander during summer (off-season) resulted in higher market price of green coriander, which ultimately reflected in higher net return and BCR. In India, green coriander is used for seasoning and flavouring of foods has become an integral part of every household, so a constant demand was observed in the market.

The intensification of seed spices-based cropping systems through legumes as components resulted in a marked improvement of organic carbon percentage as compared to initial value. This may be attributed to continuous three-year root biomass cultivation to the soil as well 300% cropping intensity [32]. Further, organic manures (FYM; 3 t ha^-1^ & vermicompost; 1.5 q ha^-1^) were also applied uniformly across the experimental site [33]. The maximum increase in organic carbon over initial value was observed in fenugreek-green coriander-clusterbean at par with fenugreek-clusterbean-clusterbean, this was closely followed by nigella-green coriander-mungbean and nigella-green coriander-clusterbean systems. This might be due to inclusion of legumes in a system could have resulted in greater organic carbon content. Kumar *et al*. [34] reported that the inclusion of a legume crop in the system increased the organic carbon and availability of N, P and K in the soil. Boone [35] reported that 16–33% of the total C assimilated by plants is released directly into the soil by roots, which contributes to 30–60% of the organic C pool in soil. The highest increase in available N over initial value was recorded in nigella-green coriander-mungbean and fenugreek-green coriander-clusterbean followed by nigella-green coriander-clusterbean cropping system. Moharana *et al*. [36] reported that the cropping system of chickpea–groundnut (legumes) maintained a higher available N content in the soils due to their ability to accumulate a notable quantum of atmospheric nitrogen in the rhizosphere. The systems having legumes showed better available nitrogen in the soil because of their nitrogen fixation ability.

Available P content decreased significantly from their initial value might be due to P adsorption and precipitation because of application of saline irrigation water during post rainy and summer season. Among the cropping system, significantly higher available P was recorded under the coriander-green coriander-clusterbean followed by fenugreek-green coriander-mungbean. Available K content was also depleted in all the cropping systems over the initial value. However, significantly higher available K content was registered under coriander-green coriander-clusterbean followed by coriander-green coriander-mungbean. Among the cropping systems nigella-clusterbean-mungbean and nigella-clusterbean-clusterbean were found most exhaustive cropping systems.

## Conclusions

Intensified seed spices-based cropping system having great potential to harness increased productivity, resource-use efficiency and livelihood. Henceforth, the seed spices growers of arid and semi-arid regions of India are recommended to adopt nigella-green coriander-mungbean cropping system in the view of better productivity, resource-use efficiency and profitability.

## Supporting information

S1 Table Prevailing weather conditions during the period of experimentation from 2019–20 to 2021–22. (PDF)

S2 Table Details of varieties and agronomic practices followed during field experimentation (2019–20 to 2021–22). (PDF)
