# Supplementary material for: Intensified seed spices-based cropping systems for higher productivity, resource-use efficiency, soil fertility and profitability in arid and semi-arid regions of India
Source: PLoS One. 2023 Oct 18;18(10):e0292955. doi: 10.1371/journal.pone.0292955 (PMC10584124; doi:10.1371/journal.pone.0292955)
Supplement: S1 Table — (PDF) [file pone.0292955.s001.pdf]

S1 Table. Prevailing weather conditions during the period of experimentation from 2019-20 to 2021-22.

Month	Minimum temperature (°C)				Maximum temperature (°C)				Evaporation (mm/day)				Rainfall (mm)			
	2019-20	2020-21	2021-22	Mean	2019-20	2020-21	2021-22	Mean	2019-20	2020-21	2021-22	Mean	2019-20	2020-21	2021-22	Total
October	17.2	25.8	18.5	20.50	33.5	32.5	33.2	33.07	6.3	11.7	5.4	7.80	8	160	10.3	178.3
November	15.2	25	10.2	16.80	29.2	34.9	29.4	31.17	4.4	6.4	4.6	5.13	0	80	3	83
December	5.8	18.3	7.4	10.50	22	35	23.7	26.90	2.4	6.4	2	3.60	10	0	0	10
January	6	6.4	6	6.13	21.6	23.5	20.7	21.93	2.2	2.8	1.2	2.07	7.1	23	21	51.1
February	6.8	8.9	8.28	7.99	27.2	30.4	28.07	28.56	4.6	4.1	3.25	3.98	0	0	0	0
March	6	15.1	20.32	13.81	21.6	35.3	34.96	30.62	2.2	9.6	7.14	6.31	7.1	3	8	18.1
April	6.8	19.8	22.2	16.27	27.2	39.3	40.65	35.72	4.6	18.8	10.54	11.31	0	0	0	0
May	16.8	24.5	27.83	23.04	29.5	38	41.11	36.20	5.2	14.4	11.93	10.51	0	29.1	25	54.1
June	20.1	26.6	28.03	24.91	37.9	38.8	38.41	38.37	11.1	14.6	9.54	11.75	25	21.1	101	147.1
July	25.2	26.9	25.61	25.90	41.9	36.5	31.41	36.60	20	9.3	2.45	10.58	17.4	161	516	694.4
August	26.6	25	25.16	25.59	39.2	33.8	30.69	34.56	16.2	7.5	2.17	8.62	56.5	197	242	495.5
September	27.1	24.7	24.63	25.48	36.7	32.9	33.58	34.39	12	3.9	4.91	6.94	131	176	0	307
Mean	14.97	20.58	18.68	18.08	30.63	34.24	32.16	32.34	7.60	9.13	5.43	7.38	262.10	850.20	926.30	2038.60
