# Supplementary material for: Intensified seed spices-based cropping systems for higher productivity, resource-use efficiency, soil fertility and profitability in arid and semi-arid regions of India
Source: PLoS One. 2023 Oct 18;18(10):e0292955. doi: 10.1371/journal.pone.0292955 (PMC10584124; doi:10.1371/journal.pone.0292955)
Supplement: S2 Table — (PDF) [file pone.0292955.s002.pdf]

S2 Table. Details of varieties and agronomic practices followed during field experimentation

Cropping system	Varieties			Seed rate kg/ha			Spacing (cm)			Rate of fertilizer application (kg/ha)								
	Rabi	Summer	Kharif	Rabi	Summer	Kharif	Rabi	Summer	Kharif	Rabi			Summer			Kharif		
										N	P	K	N	P	K	N	P	K
CS_1	ACr-1	RGC-1038	SML-668	12	15	12	20x15	45x15	40x15	40	30	20	30	48	0	30	48	0
CS_2	ACr-1	RGC-1038	RGC-1038	12	15	15	20x15	45x15	45x15	40	30	20	30	48	0	30	48	0
CS_3	ACr-1	AGCr-1	RGC-1038	12	12	15	20x15	20x15	45x15	40	30	20	600	400	200	30	48	0
CS_4	ACr-1	AGCr-1	SML-668	12	12	12	20x15	20x15	40x15	40	30	20	600	400	200	30	48	0
CS_5	AFg-3	RGC-1038	SML-668	20	15	12	25x10	45x15	40x15	25	20	20	30	48	0	30	48	0
CS_6	AFg-3	RGC-1038	RGC-1038	20	15	15	25x10	45x15	45x15	25	20	20	30	48	0	30	48	0
CS_7	AFg-3	AGCr-1	RGC-1038	20	12	15	25x10	20x15	45x15	25	20	20	600	400	200	30	48	0
CS_8	AFg-3	AGCr-1	SML-668	20	12	12	25x10	20x15	40x15	25	20	20	600	400	200	30	48	0
CS_9	AN-20	RGC-1038	SML-668	6	15	12	30x15	45x15	40x15	40	20	20	30	48	0	30	48	0
CS_{10}	AN-20	RGC-1038	RGC-1038	6	15	15	30x15	45x15	45x15	40	20	20	30	48	0	30	48	0
CS_{11}	AN-20	AGCr-1	RGC-1038	6	12	15	30x15	20x15	45x15	40	20	20	600	400	200	30	48	0
CS_{12}	AN-20	AGCr-1	SML-668	6	12	12	30x15	20x15	40x15	40	20	20	600	400	200	30	48	0

CS_1-coriander-clusterbean-mungbean, CS_2-coriander-clusterbean-clusterbean, CS_3-coriander-green coriander-clusterbean, CS_4-coriander-green coriander-mungbean, CS_5-fenugreek-clusterbean-mungbean, CS_6-fenugreek-clusterbean-clusterbean, CS_7-fenugreek-green coriander-clusterbean, CS_8- fenugreek-green coriander-mungbean, CS_9-nigella-clusterbean-mungbean, CS_{10}-nigella-clusterbean-clusterbean, CS_{11}-nigella-green coriander-clusterbean, CS_{12}-nigella-green coriander-mungbean
